# An endogenous factor enhances ferulic acid decarboxylation catalyzed by phenolic acid decarboxylase from *Candida guilliermondii*

**DOI:** 10.1186/2191-0855-2-4

**Published:** 2012-01-04

**Authors:** Hui-Kai Huang, Li-Fan Chen, Masamichi Tokashiki, Tadahiro Ozawa, Toki Taira, Susumu Ito

**Affiliations:** 1 United Graduate School of Agricultural Sciences, Kagoshima University, Kagoshima, Kagoshima 890-0065, Japan; 2 Department of Bioscience and Biotechnology, University of the Ryukyus, Nishihara, Okinawa 903-0213, Japan; 3 Tochigi Research Laboratories of Kao Corporation, Ichikai, Haga, Tochigi 321-3497, Japan

**Keywords:** phenolic acid decarboxylase, ferulic acid decarboxylase, *p*-coumaric acid decarboxylase, *Candida guilliermondii*, activator

## Abstract

The gene for a eukaryotic phenolic acid decarboxylase of *Candida guilliermondii *was cloned, sequenced, and expressed in *Escherichia coli *for the first time. The structural gene contained an open reading frame of 504 bp, corresponding to 168 amino acids with a calculated molecular mass of 19,828 Da. The deduced amino sequence exhibited low similarity to those of functional phenolic acid decarboxylases previously reported from bacteria with 25-39% identity and to those of PAD1 and FDC1 proteins from *Saccharomyces cerevisiae *with less than 14% identity. The *C. guilliermondii *phenolic acid decarboxylase converted the main substrates ferulic acid and *p*-coumaric acid to the respective corresponding products. Surprisingly, the ultrafiltrate (Mr 10,000-cut-off) of the cell-free extract of *C. guilliermondii *remarkably activated the ferulic acid decarboxylation by the purified enzyme, whereas it was almost without effect on the *p*-coumaric acid decarboxylation. Gel-filtration chromatography of the ultrafiltrate suggested that an endogenous amino thiol-like compound with a molecular weight greater than Mr 1,400 was responsible for the activation.

## Introduction

Ferulic acid (FA), a derivative of 4-hydroxycinnamic acid, is found in cell walls primarily as an ester linked to lignin and other polysaccharides in cell walls, leaves and seeds of plants such as in rice, wheat, and oat [Mathew and Abraham 2004]. Bacterial phenolic acid decarboxylases (PADs), which decarboxylate FA, *p*-coumaric acid (PCA), and/or caffeic acid (CA) with concomitant production of 4-vinylguaiacol (4VG), 4-vinylphenol (4VP), and/or 4-vinylcatechol, respectively (see Additional file 1), are responsible for the detoxification of these 4-hydroxycinnamic acids [Huang et al. 1994,Degrassi et al. 1995,Cavin et al. 1997b,^1998^]. [Zago et al. (1995)] first succeeded in sequencing and expression of a bacterial PAD (FA decarboxylase from *Bacillus pumilus*) in *Escherichia coli*. The genetic mechanism of bacterial PAD expression has been well established by the discovery of PadR-mediated response to 4-hydroxycinnamic acids in *Pediococcus pentosaceus *[Barthelmebs et al. 2000], *Bacillus subtilis *[Tran et al. 2008], and *Lactobacillus plantarum *[Gury et al. 2009]. The 4VG formed is valuable precursor in the biotransformation of flavors and fragrances used in the food, pharmaceutical, and cosmetic industries [Mathew and Abraham 2006,Priefert et al. 2001]. Furthermore, this compound is sometimes present as an aroma in beers and wines [Thurston and Tubb 1981,Smit et al. 2003,Coghe et al. 2004,Oelofse et al. 2008,Sáez et al. 2010]

Naturally-occurring phenolic acids are known to inhibit the growth of yeasts such as *Saccharomyces cerevisiae, Pichia anomala, Debaryomyces hansenii*, and *Candida guilliermondii *(*Meyerozyma guilliermondii *comb. nov.; [Kurtzman and Suzuki 2010]) [Baranowski et al. 1980,Stead 1995,Pereira et al. 2011]. *S. cerevisiae *[Goodey and Tubb 1982,Clausen et al. 1994,Smit et al. 2003,Coghe et al. 2004], *Brettanomyces bruxellensis *[Godoy et al. 2008], and *C. guilliermondii *[Huang et al. 2011] are suggested to produce a PAD in response or relation to 4-hydroxycinnamic acids.

Recently, we purified and characterized a highly active substrate-inducible PAD from *C. guilliermondii *ATCC 9058 (CgPAD) [Huang et al. 2011]. CgPAD is heat-labile, and its molecular mass determined by SDS-polyacrylamide gel electrophoresis is about 20 kDa, which is similar to those of yeast strains of *Brettanomyces anomalus *[Edlin et al. 1998] and *B. bruxellensis *[Godoy et al. 2008]. CgPAD was active toward 4-hydroxycinnamic acid derivatives, PCA, FA, and CA, whose relative activity ratios are different from the PADs of *B. anomalus *and *B. bruxellensis*.

In the case of *C. guilliermondii *ATCC 9058, CgPAD may be induced by both PCA and FA, because the ratios of decarboxylation activity toward FA to PCA in the cell-free extracts were comparable to that of the purified enzyme. However, 6-hydroxy-2-naphthoic acid (6H2N) induced CgPAD 20- and 6-fold greater than FA and PCA, respectively, and the ratios of decarboxylation activity toward FA to PCA in the cells grown on different carbon sources in the presence of the pseudo-inducer were found to be increased remarkably [Huang et al. 2011]. There was a possibility that 6H2N induced another FA decarboxylase distinct from CgPAD under a defined condition, but such activity was not detectable during the course of purification. In the present study, to resolve this inconsistency, we sequenced the gene for CgPAD and created recombinant enzymes. Unexpectedly, we found that the presence of dithiothreitol (DTT), 2-mercaptoethanol, cysteine, and homocysteine considerably accelerated the rates of FA decarboxylation activity of the purified native and recombinant CgPAD, while they did not affect those of their PCA decarboxylation activity. We also demonstrated that an unidentified amino thiol-like compound in the ultrafiltrate of the *C. guilliermondii *cell-free extract enhanced the FA decarboxylation activity specifically.

## Materials and methods

### Materials

FA, CA, 4VG, and 6H2N were purchased from Wako Pure Chemical (Osaka, Japan). PCA was from MP Biomedicals (Solon, OH), and 4VP was from Sigma-Aldrich (Steinheim, Germany). All other chemicals used were of analytical grade.

### Microorganisms and propagation

The source of PAD and its gene was *C. guilliermondii *(*M. guilliermondii*) ATCC 9058. The enzyme was induced aerobically by 6H2N (1 mM) in Yeast Nitrogen Base (YNB; Invitrogen, Carlsbad, CA) broth containing 0.5% glucose as described [Huang et al. 2011]. Briefly, the yeast was grown at 25°C for 1 d, with shaking, in 200-ml portions of the medium placed in 2-l flasks. *E. coli *DH5α (Takara Bio, Otsu, Japan) and *E. coli *BL21 (DE3) (Takara Bio) were used for plasmid preparation and sequencing and for expression and purification of recombinant CgPAD, respectively. The transformed *E. coli *cells were grown, with shaking, at 37°C in 50-ml portions of Luria-Bertani broth plus ampicillin (100 μg ml^-1^) placed in 500-ml flasks to an *A*_600 _of 0.5. After adding isopropyl β-D-galactosyl pyranoside (0.1 mM) to the culture, incubations were further continued at 18°C for 24 h. After cells were collected by centrifugation (12,000 × *g *for 10 min) at 4°C, cell pastes obtained from 600-ml culture were used as the starting materials for enzyme purification.

### Purification of native and recombinant forms of CgPAD

Enzyme purification was done at a temperature not exceeding 4°C. The native CgPAD in *C. guilliermondii *was purified by successive column chromatographies on CM Toyopearl 650 M (Tosoh, Tokyo, Japan), DEAE Toyopearl 650 M (Tosoh), and Bio-Gel P-100 (Bio-Rad, Hercules, CA) columns, as described previously [Huang et al. 2011].

The wild-type and mutant recombinant enzymes highly expressed in *E. coli *cells were each purified by essentially the same procedure as that of the native enzyme [Huang et al. 2011]. The recombinant *E. coli *cells were washed twice with saline and then suspended in two volumes of the extraction buffer [20 mM sodium phosphate buffer (pH 7.0) plus 1 mM each of phenylmethanesulfonyl fluoride, MgCl_2_, EDTA, and DTT]. The cells were disrupted six times for 50 s each with glass beads (0.5 mm in diameter) at 2,500 rpm in a homogenizer (Multi-Beads Shocker; Yasui Kikai, Osaka, Japan). After cell debris was removed by centrifugation (12,000 × *g*, 15 min), the supernatant obtained was applied directly to a column of DEAE Toyopearl 650 M (2.5 cm × 25.5 cm) previously equilibrated with 20 mM 2-morpholinoethanesulfic acid/NaOH (MES) buffer (pH 6.5). The column was initially washed with 200 ml of 50 mM NaCl in MES buffer (pH 6.5), and proteins were eluted with a 300-ml linear gradient of 50 mM to 0.5 M NaCl in the buffer. The active fractions were immediately concentrated and exchanged with 50 mM phosphate buffer (pH 7.0) by ultrafiltration (Amicon Ultra-15; Millipore, Billerica, MA) to a small volume. The concentrate was then put on a column of Bio-Gel P-100 (1.0 cm × 43 cm) equilibrated with 50 mM sodium phosphate buffer (pH 7.0) and eluted with the equilibration buffer. The active fractions were combined and concentrated by ultrafiltration, and the concentrate was stored at -20°C until use. Highly purified wild-type and mutant recombinant enzymes were obtained approximately 2- to 5-folds with yields of 40-70% within 2 d by the simple purification procedure as judged by SDS-acrylamide gel electrophoresis (see Additional file 2).

### Assay of CgPAD activity

The enzyme assay method was essentially the same as described previously [Huang et al. 2011]. The initial velocity of decarboxylation activity was measured at 25°C with 4-hydroxycinnamic acid as substrate. The reaction mixture contained the suitably-diluted enzyme solution and a 5 mM substrate (neutralized with 1.0 N NaOH) in 0.1 M sodium phosphate buffer (pH 6.0) in a final volume of 1.0 ml. After the reactions were terminated by boiling for 10 min, the products formed were quantified by high-performance liquid chromatography (HPLC) using a packed column for reversed phase chromatography (Cosmosil 5C18-MS-II, 4.6 mm × 150 mm; Nacalai Tesque, Tokyo, Japan) with acetonitrile/0.05% phosphoric acid (7:3, v/v) as the mobile phase at a flow rate of 0.6 ml min^-1^. One U of enzyme activity was defined as the amount of enzyme that released 1 μmol of 4VG or 4VP per min. Because the product, 4-vinylcatechol, from CA was not commercially available, the CA decarboxylation activity was expressed as formation of 4VG. Protein concentrations were measured using a BCA protein assay kit (Thermo Fisher Scientific, Rockville, MD) with bovine serum albumin as the standard.

### Sequencing of internal amino acid residues of CgPAD

Initially, peptides of native CgPAD were obtained by treatment with CNBr or *Staphylococcus aureus *V8 protease. The CNBr cleavage was done essentially by the method of [Steers et al. (1965)]. One mg of CgPAD was dissolved in 0.2 ml of 70% formic acid and cleaved with an excess of CNBr at room temperature for 24 h. After the remaining CNBr was removed by a rotary evaporator, the reaction mixture was filtered on a column of TSK gel G2000SWXL (Tosoh, 0.78 cm × 30 cm) in 30% acetic acid. The digestion of CgPAD with V8 protease was performed at 37°C for 6 h in 50 mM ammonium bicarbonate buffer (pH 7.8) plus 4 M urea and 2 mM EDTA. The peptide fragments obtained were fractionated by reverse-phase high-performance liquid chromatography on a C4 column (3.9 mm × 150 mm, Waters, Milford, MA). The amino acid sequences of peptides derived from CNBr or V8 protease digestion of native CgPAD were determined by automated sequential Edman degradation using a PPSQ-23A protein sequencing system (Shimadzu, Kyoto, Japan).

### Cloning and sequencing of CgPAD gene

All primers used are presented in Additional file 3. By a reverse transcription polymerase chain reaction using appropriate degenerate primers, the internal cDNA fragments of CgPAD were sequenced. Total RNA was isolated from *C. guilliermondii *using an RNeasy kit (Qiagen, Valencia, CA). First-strand cDNA synthesis was performed with 5 μg of total RNA using a GeneRacer kit (Invitrogen, Carlsbad, CA) with oligo(dT) adaptor primer. The cDNA obtained was used as a template for PCR amplification with degenerate primers. The first PCR was performed with primers P1 (a forward primer designed from an internal LKNKHFQYTYDNGWKYEFHV) and P2 (a reverse primer designed from an internal AFSQGHWEHPEQAHGDKRED), and nested PCR was done with P1 and P3 (a reverse primer designed from an internal AFSQGHWEHPEQAHGDKRED) (the sequences used for primer design are underlined). The nested PCR product was then cloned into a pGEM-T vector (Promega, Madison, WI) and sequenced using the ABI Prism system (Model 310; Applied Biosystems, Foster City, CA).

To obtain the entire gene for CgPAD, both 5' and 3' rapid amplification of cDNA ends (RACE) were performed using a GeneRacer kit according to the manufacturer's instructions. The gene-specific primers P4 (first PCR) and P5 (nested PCR) were used for the 5'-RACE and the gene-specific primers P6 (first PCR) and P7 (nested PCR) for the 3'-RACE. Approximately 250 bp and 290 bp were amplified by 5'-RACE and 3'-RACE, respectively. Finally, a cDNA fragment containing the entire coding region of CgPAD cDNA was amplified using the forward primer P8 (designed from the 5'-RACE product) and reverse primer P9 (designed from the 3'-RACE product).

The nucleotide sequence of CgPAD was submitted to DDBJ under the accession number AB663499

### Site-directed mutagenesis

The entire CgPAD gene was amplified by PCR and cloned into the *Nde*I/*Hin*dIII site of pET-22b (+) (Novagen, Darmstadt, Germany), yielding the construct designated pPAD22b. Amino acid replacements were performed using a QuikChange II site-directed mutagenesis kit (Stratagene, La Jolla, CA). PCR was performed using PfuUltra HF DNA polymerase (Promega) with pPAD22b as the template. The primer sets used were 5'-CATGGGGGGCCACTGGCTGGACGGCAC-3'/5'-GTGCCGTCCAGCCAGTGGCCCCCCATG-3' for the Met57→Leu (M57L) mutation, 5'-CATGGGGGGCCAACGGCTGGACGGCAC-3'/5'-GTGCCGTCCAGCCGTTGGCCCCCCATG-3' for the M57T mutation, and 5'-CATGGGGGGCCAGCGGCTGGACGGCAC-3'/5'-GTGCCGTCCAGCCGCTGGCCCCCCATG-3' for the M57A mutation (the underlined sequences indicate mutated codons). To express mutant proteins, the resulting plasmids harboring the respective mutated genes were each introduced into competent *E. coli *BL21 (DE3) cells.

### Construction of model structure of CgPAD

The secondary structure of CgPAD was predicted by the method of [Kabsch and Sander (1983)]. The deduced amino acid sequence of the enzyme was aligned with that of the crystal structure of a PAD (PCA decarboxylase) from *L. plantarum *(LpPAD; PDB code 2GC9) [Rodríguez et al. 2010]. A model of the CgPAD structure built with method of homology modeling was constructed based on the structure of LpPAD [Sali et al. 1993]. All data sets were processed on a Windows XP personal computer using the Discovery Studio software package (Accelrys, San Diego, CA). Distance between intramolecular sulfur atom of methionine and side-chain carbonyl oxygen atoms of glutamic acid or amide nitrogen atom of arginine was calculated from the coordinate values. The figure was prepared using a DS Visualizer (Accelrys).

## Results

### Nucleotide and deduced amino acid sequences of CgPAD

**Figure 1:**
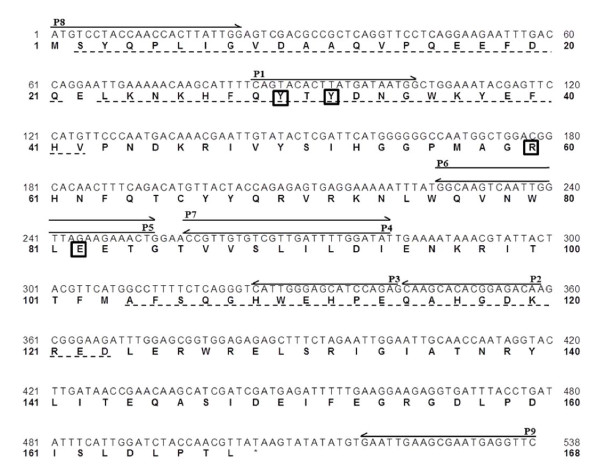
**Nucleotide and deduced amino acid sequences of CgPAD**. The primers used for cloning the CgPAD gene are indicated by arrows above the nucleotide sequence. Dotted underlines indicate deduced amino acid sequences identical to those of the peptides derived from CNBr and *S. aureus *V8 protease digestions of native CgPAD. The residues possibly involved in the catalysis, which are integrally conserved in LpPAD [Rodríguez et al. 2010], are boxed.

We initially cloned and sequenced the CgPAD gene. The entire CgPAD gene was 504 nucleotides in length, and an open reading frame encoded 168 amino acid residues (Figure 1). The calculated molecular mass was 19,828 Da http://web.expasy.org/compute_pi/, a value very close to the 20 kDa determined for the native enzyme by SDS-polyacrylamide gel electrophoresis [Huang et al. 2011].

**Figure 2:**
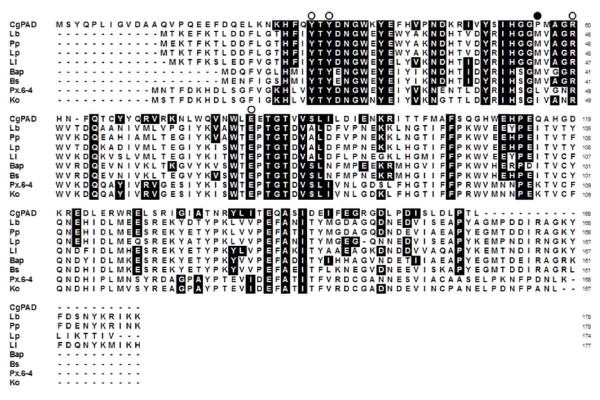
**Multiple amino acid sequence alignment of CgPAD with functional PADs reported to date**. The sequences used are *L. brevis *ATCC 367 (RM84) (Lb; ABJ63379), *P. pentosaceus *ATCC 25745 (Pp; AJ276891), *L. plantarum *WCFS1 (Lp; PDB code 2GC9; gi93279967), *L. plantarum *LPCHL2 (Ll; U63827), *B. pumilus *PS213 (*Bap*; X84815), *B. subtilis *168 (Bs; AF017117), *Enterobacter *sp. Px6-4 (Px6-4; ACJ26748), and *K. oxytoca *(Ko; BAF65031). The sequences were aligned using the ClustalW program. Residues identical to those in CgPAD are shaded. Possible catalytic residues are indicated as open circles above the CgPAD sequence. Partially conserved Met residues are indicated as filled circle. Sequences of homologous hypothetical proteins of unknown function in the reported genome sequences are not included in the multiple alignments.

The deduced amino acid sequence of CgPAD was aligned with those of functional PADs reported to date from different bacteria [Thompson et al. 1997]; http://www.genome.jp/tools/clustalw). As shown in Figure 2, CgPAD exhibited very low similarity of sequence to functional PADs reported to date from *L. plantarum *WCFS1 ([Rodríguez et al. 2010] and *L. plantarum *LPCHL2 [Cavin et al. 1997a] with 39%, *Enterobacter *sp. Px6-4 [Gu et al. 2011a,2011b] with 34%, *Klebsiella oxytoca *[Uchiyama et al. 2008] with 27%, and *B. subtilis *168 [Cavin et al. 1998] with 26% identity, and *B. pumilus *PS213 [Zago et al.1995], *P. pentosaceus *ATCC 25745 [Barthelmebs et al. 2000], and *Lactobacillus brevis *ATCC 367 (RM84) [Landete et al. 2010] each with 25% identity. Nevertheless, four residues (Tyr18, Tyr20, Arg48, and Glu71) involved in the catalysis of LpPAD [Rodríguez et al. 2010] were well conserved in CgPAD as Tyr30, Tyr32, Arg60 [but Asn23 in the *Enterobacter *enzyme [Gu et al. 2011b]], and Glu82 and of PADs from other bacteria as these residues at the corresponding positions.

### Construction of model structure and creation of mutant proteins of CgPAD

The absence and/or replacement of methionine residues adjacent to catalytic residues or in the proximate area of active-site pockets has been reported to confer resistance to oxidation, as based on the catalytic activity of enzymes [Estell et al. 1985,Hagihara et al. 2001,^2003^,Nonaka et al. 2003]. Further, we demonstrated that the replacement or oxidation of such the Met residues altered the conversion rates of substrates by some enzymes [Hagihara et al. 2001,^2003^,Nonaka et al. 2003,^2004^,Saeki et al. 2007]. Then, we first postulated that the fluctuation of the ratio of decarboxylation toward FA to PCA might result from oxidation of heat-labile CgPAD, because the deduced amino acid sequence contained two oxidizable Met residues at positions 57 and 103 (Figure 1). It was expected that the replacement of either Met57 or Met 103 with the non-oxidizable amino acids would increase the ratio of decarboxylation activity of CgPAD toward FA to PCA.

According to this scenario, we constructed a model of CgPAD using the crystal structure of LpPAD (PDB code 2GC9) as the template. In the result, the Met residues at positions 57 and 103 in the modeled CgPAD appeared to be located in the active-site pocket (see Additional file 4). Especially, the Met57 residue is located at the entrance of the pocket and in the immediate vicinity of possible catalytic residues Arg60 and Glu82. However, the Met103 residue is spatially more distant from the two catalytic residues and located deeper in the active-site pocket (see Additional file 5).

Therefore, we selected the Met57 residue as the target for site-directed mutagenesis and created the mutant enzymes with M57L, M57T, and M57A. The recombinant wild-type and mutant enzymes expressed in *E. coli *cells, together with the native enzyme from *C. guilliermondii*, were each purified to homogeneity (see Additional file 2). Contrary to our expectation, a mutant enzyme with M57L did not increase the ratio of decarboxylation activity of CgPAD toward FA to PCA compared with the native and recombinant wild-type enzymes, as shown in Table 1. The activities toward both substrates of the mutants with M57T and M57A were practically negligible.

**Table 1:** Substrate specificities of native and recombinant enzymes

Enzyme	Substrate	Specific activity (U mg^-1^)	Relative activity ^a ^(%)
Native	FA	378 ± 16	100
	PCA	393 ± 6	104
	CA	53 ± 9	14

Recombinant	Wild-type		
	FA	260 ± 30	100
	PCA	259 ± 4.0	100
	CA	31.7 ± 2.8	12
	M57L		
	FA	20.1 ± 0.3	100
	PCA	20.5 ± 0.3	102
	CA	2.93 ± 0.03	15
	M57T		
	FA	< 0.1	-
	PCA	< 0.1	-
	CA	< 0.01	-
	M57A		
	FA	< 1.0	-
	PCA	< 1.0	-
	CA	< 0.02	-

^a ^The FA decarboxylation activities of the native and recombinant enzymes are taken as 100% for each.

### Acceleration of FA decarboxylation activity of CgPAD by thiol compounds

**Figure 3:**
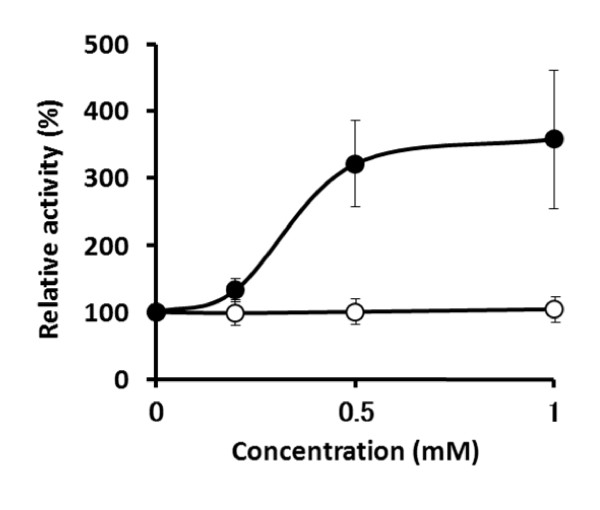
**Effect of DTT on decarboxylation activities toward FA and PCA of purified native CgPAD**. DTT (0.2, 0.5, or 1 mM) was added to the reaction mixtures containing ×10 enzyme (1.0-ml final volume) and incubated at 25°C for 20 min. An aliquot (0.1 ml) was withdrawn and added to 0.9 ml of the reaction mixture containing 0.1 M FA or PCA, and the initial velocities of decarboxylation of FA (filled circle) and PCA (open circle) were measured under the standard conditions of enzyme assay. The values obtained from three separate experiments are shown and expressed as percentages, taking the activity of each untreated enzyme as 100%. The bars indicate the standard deviation at each point.

For measurement of enzyme activities in the cell-free extracts, we disrupted the cells in the extraction buffer supplemented with 1 mM DTT. Then, we examined the effect of this thiol on the activity of purified native CgPAD. As the result, the FA decarboxylation activity was found to be enhanced by DTT at 0.2-1 mM (Figure 3), while the PCA decarboxylation activity was not affected by DTT at the concentrations examined.

To further understand the unexpected positive effect of DTT, we examined the effects of various thiol-containing amino acids and chemical reagents on both activities. Positive effects on the FA decarboxylation activity were also observed with 2-mercaptoethanol (5 mM) and sulfhydryl amino acids such as L-cysteine, D-cysteine, and DL-homocysteine (1 mM each), and the increases in the relative decarboxylation activities of FA to PCA reached 2:1 to 3:1 when compared with the control (without thiol), as shown in Table 2. Cysteic acid (1 mM) was essentially without effect.

**Table 2:** Effects of thiol reagents on the activities of native CgPAD

Additive (1 mM)	Specific activity (U mg^-1^)		FA/PCA
		
	FA (%)	PCA (%)	
None	378 ± 16 (100)	393 ± 6 (100)	0.96
2-Mercaptoethanol (5 mM)	806 ± 56 (213)	440 ± 3 (112)	1.83
Dithiothreitol	1140 ± 177 (302)	598 ± 7 (112)	1.91
L-Cysteine	1130 ± 37 (299)	437 ± 39 (111)	2.59
D-Cysteine	1590 ± 252 (421)	590 ± 4 (150)	2.69
DL-Homocysteine	1830 ± 354 (484)	628 ± 9 (160)	2.91
Cysteic acid	557 ± 30 (147)	593 ± 9 (151)	0.94

Experimental conditions were the same as those described in Figure 3.

The possibility that the replacement of Met57 with leucine disrupted the structural proper folding of CgPAD was not excluded because its specific activity was considerably lower than those of the native and wild-type enzymes (Table 1). However, the positive effect by L-cysteine (1 mM) on FA decarboxylation activity was also observed with the M57L mutant enzyme as well as with native and recombinant wild-type forms, as shown in Table 3. Essentially, L-cysteine was without effect on the decarboxylation activity toward CA of the wild-type enzyme. The activity toward CA of the M57L mutant enzyme was too low to evaluate the effect of L-cysteine.

**Table 3:** Effect of L-cysteine on the activities of recombinant wild-type and M57L mutant CgPAD

Substrate	Specific activity (U mg^-1^)	Relative activity ^b ^(%)
Wild type		
FA	260 ± 9.0	100
FA + L-cysteine ^a^	759 ± 156	292
PCA	259 ± 4.0	100
PCA + L-cysteine	257 ± 3.3	99
CA	31.7 ± 2.8	12
CA + L-cysteine	35.9 ± 3.5	14
M57L		
FA	20.1 ± 0.3	100
FA + L-cysteine ^a^	66.7 ± 8.5	332
PCA	20.5 ± 0.3	100
PCA + L-cysteine	26.0 ± 2.7	127
CA	< 2.6	< 13
CA + L-cysteine	ND ^c^	

Experimental conditions were the same as those described in Figure 3. ^a ^Treated with 1 mM. ^b ^The activity toward FA is taken as 100%. ^c ^Not determined.

### Activation of FA decarboxylation activity by ultrafiltrate of cell-free extract

**Figure 4:**
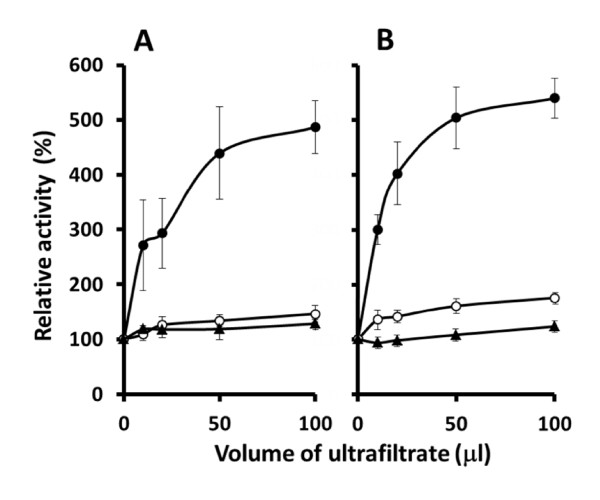
**Effect of ultrafiltrate on activities of native (A) and recombinant wild-type CgPAD (B)**. Cell pastes of 6H2N-induced *C. guilliermondii *and recombinant *E. coli *were disrupted with glass beads in the extraction buffer. The fresh cell-free extracts obtained (5.8-7.5 mg protein ml^-1^) were ultrafiltered by centrifugation in an Amicon Ultra-15. Aliquots (10 μl to100 μl) of the ultrafiltrate were added immediately to the reaction mixture containing enzyme (0.95-ml final volume), incubated at 25°C for 20 min, and then the initial velocities toward substrates were measured by adding 50 μl of a 0.1 M substrate. The values were obtained from several separate experiments and are expressed as percentages, taking the activity toward FA (filled circle), PCA (open circle), and CA (filled triangle) of the untreated enzyme as 100%.

Finally, we supposed that *C. guilliermondii *inherently possessed a physiological thiol activator, which might have been removed during the enzyme purification. Then, we prepared an ultrafiltrate of the cell-free extract (Mr 10,000-cut-off) of induced *C. guilliermondii *cells and incubated it with native and recombinant CgPADs at 25°C for 20 min before enzyme assays. As the results shown in Figure 4, the ultrafiltrate was found to remarkably increase the FA decarboxylation activities of both enzymes (approximately up to 5-folds) with an increase in its volume, while it exhibited little effect on their PCA and CA decarboxylation activities. The ultrafiltrate of the cell-free extract of recombinant *E. coli *did not exhibit such an activation effect.

### Partial purification of true activator in the ultrafiltrate

**Figure 5:**
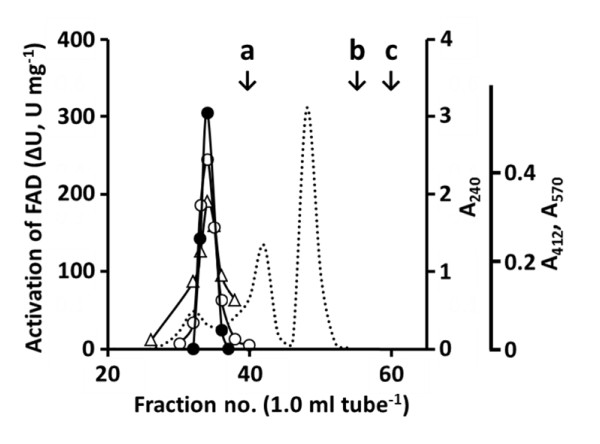
**Gel-filtration chromatography of ultrafiltrate**. One ml of the ultrafiltrate prepared from the cell-free extract of induced *C. guilliermondii *cells was applied to a Bio-Gel P-2 column (1.0 cm × 50 cm) previously washed with distilled water and eluted with distilled water. The column was calibrated with cyanocobalamin (a, Mr = 1,355), cellobiose (b, Mr = 342), and glucose (c, Mr = 180), as indicated by vertical arrows. The elution pattern was monitored by measuring at 240 nm (dotted line). The fractions containing the activator of FAD activity (filled circle) were examined for color reactions with DTNB solution at 412 nm (open circle) and ninhydrin solution at 570 nm (open triangle). The activation was expressed as ΔU mg^-1 ^[(activity + eluate)-(control activity)] after 20-min incubation at 25°C. For the color reactions, 0.1-ml aliquots were added to 0.9 ml of 5 mM DTNB in 50 mM phosphate buffer (pH 7.5) and ninhydrin solution, respectively.

The ultrafiltrate was subjected to gel-filtration chromatography on Bio-Gel P-2. As shown in Figure 5, a possible true activator associated with the FA decarboxylation activity was detected in fractions corresponding to a Mr larger than 1,400. The fractions reacted positively with the 5,5'-dithio-bis(2-nitrobenzoic acid) (DTNB) and ninhydrin reagents. In the presence of 50 μl of the filtrate, the values of *K*_m_, *k*_cat_, and *k*_cat_/*K*_m _for FA were 5.67 mM, 278 s^-1^, and 49.0 s^-1 ^mM^-1^, whereas those of the control (without filtrate) were 5.31 mM, 89.7 s^-1^, and 16.9 s^-1 ^mM^-1^, respectively.

## Discussion

This study describes for the first time the cloning, sequencing, and expressing the gene for a eukaryotic PAD in *E. coli*. CgPAD exhibited very low sequence similarity to reported functional PADs with 24-39% identity. CgPAD showed 100% amino acid sequence identity to a hypothetical protein (EDK35930; locus tag PGUG_00028) in the genome of the yeast *M. guilliermondii *ATCC 6260 (AAFM00000000), and moderate similarity (51-56% identity) to the internal sequences of hypothetical proteins of unknown function in the genomes of fungi including the genera *Verticillium, Neosartorya, Aspergillus, Schizophyllum, Ustilago, Sporisorium, Nectria, Gibberella*, and *Penicillium *(data not shown). Notably, CgPAD exhibited sequence similarity to PAD1 (YDR538W) with less than 14% identity and essentially no homology with FDC1 (YDR539W) isolated from *S. cerevisiae *[Clausen et al. 1994,Mukai et al. 2010].

There was a possibility that either Met57 or Met103 in CgPAD was located in the active-site pocket and oxidized to methionine sulfoxide during growth or purification. Accordingly, the massive sulfoxide group of the oxidized Met residue in the CgPAD might hinder the entry of FA (4-hydroxy-3-methoxycinnamic acid) due to its 3-methoxy group, but not PCA (4-hydroxycinnamic acid), to the active-site pocket. To understand the fluctuation of the ratio of decarboxylation toward FA to PCA of CgPAD, we constructed a model structure of the enzyme and replaced the Met57 residue located at the entrance of the pocket with non-oxidizable amino acids by site-directed mutagenesis. However, a mutant enzyme (M57L) did not increase the decarboxylation ratio of FA to PCA, for instance. This may exclude the possibility that oxidation of Met57, close to the catalytic residues Arg60 and Glu82, alters the decarboxylation ratio of FA to PCA. A single Cys residue at position 66 could be responsible for the alteration of CgPAD activity. However, in the model of CgPAD we built, the Cys66 residue is located deeper in the active-site pocket and faced on the other side and far distant (7.2 Å) from the indole ring of Trp80 which might interact with Glu82 (data not shown). Essentially, conversion of cysteine to cysteic acid during purification steps is unlikely because cysteine is oxidized by strong chemical oxidants.

In this study, the activities toward substrates of the M57T and M57A mutants, together with M57L mutant, were found to be much lower than those of the native and recombinant wild-type forms of CgPAD. This result suggests that Met57 is one of the substrate-binding residues in the catalysis of CgPAD. In support of our view, one of substrate-binding residues, Leu45, in the *Enterobacter *PAD [Gu et al. 2011b] is conserved as Met at the corresponding positions in the aligned bacterial enzymes. The corresponding residue in CgPAD is Met57 in the model structure of CgPAD (see Additional file 4).

Unexpectedly, we found that the rate of FA decarboxylation activity, but not PCA decarboxylation activity, of CgPAD was accelerated by DTT, 2-mercaptoethanol, cysteine (both L- and D-forms), and DL-homocysteine, which are antioxidants and/or reducing reagents. However, these chemical reagents cannot reduce oxidized Met residues (methionine sulfoxide and methionine sulfonate) in protein molecules. Furthermore, L-cysteine and L-homocysteine are involved in the trans-sulfurization of amino acid metabolism (e.g., [Brosnan and Brosnan 2006]), both of which intracellular concentrations are very lowered by strict regulatory control. These results exclude the possibility that these amino acids are the physiological activator for CgPAD.

Finally, we found that an amino thiol-like endogenous factor in the ultrafiltrate of the *C. guilliermondii *cell-free extract drastically enhanced the FA decarboxylation activity. The kinetic data indicate that the ultrafiltrate increases the maximal activity toward FA without altering of the affinity to the substrate. These findings led us to conclude that a true activator for FA decarboxylation activity is inherently present in the *C. guilliermondii *cells. This also shows that the true activator was removed during the enzyme purification. Such a catalytic nature has never been reported in the literature. Identification of the structure of the endogenous activator would explain the novel catalytic feature of CgPAD and contribute to the clarification of physiological role of PADs in some yeast cells. It is interesting to examine whether such activation of eukaryotic PADs is observed by ultrafiltrates of prokaryotes and vice versa.

[Rodríguez et al. (2010)] clarified by site-directed mutagenesis of Arg48 and Glu71 in LpPAD that the entrance region, particularly the β1-β2 and β3-β4 loops, adopted a distinct closed conformation that decreased the opening of the active-site cavity. Possible subsite residues Tyr30 and Tyr32 and catalytic residue Glu82 along with Met57 of CgPAD are located on the β1-β2 loop and β3-β4 loop, respectively (see Additional file 4 A). It is possible that the physiological activator in the ultrafiltrate and/or the tested thiol compounds induce conformational change of the loops so that the entry of FA is much easier than those of PCA and CA. CgPAD exhibits low sequence similarity to LpPAD of known structure, and we are now crystalizing CgPAD to solve its X-ray structure.

## Competing interests

The authors declare that they have no competing interests.

## Supplementary Material

Additional file 1 **Reaction scheme for CgPAD with different substrates**. Supplementary scheme 1. Click here for file

Additional file 2 **SDS-polyacrylamide gel electrophoresis of purified enzymes**. Supplementary figure 1. Click here for file

Additional file 3 **Primers for CgPAD cDNA cloning**. Supplementary table 1. Click here for file

Additional file 4 **A**. Structure-based amino acid sequence alignment of CgPAD with LpPAD of known structure (PDB code 2GC9). **B**. A model structure of CgPAD incorporating possible catalytic residues Glu82 and Arg60 residues and subsite residues Tyr30 and Tyr32, along with Met57 and Cys66. Supplementary figure 2. Click here for file

Additional file 5 **A**. A CPK model of CgPAD. **B**. Coordination of the catalytic residues in the surrounding Met57 (upper) and Met103 (lower) in the model CgPAD structure. Supplementary figure 3. Click here for file
