# Supplementary material for: An endogenous factor enhances ferulic acid decarboxylation catalyzed by phenolic acid decarboxylase from Candida guilliermondii
Source: AMB Express. 2012 Jan 4;2:4. doi: 10.1186/2191-0855-2-4 (PMC3402150; doi:10.1186/2191-0855-2-4)
Supplement: Additional file 1 — Reaction scheme for CgPAD with different substrates. Supplementary scheme 1. [file 2191-0855-2-4-S1.PDF]

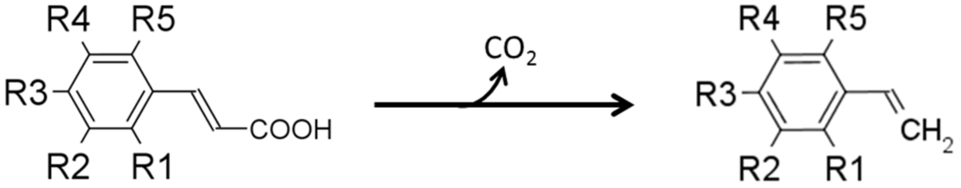

Substrate **Product**

Ferulic acid (R1=R4=R5=H, R2=OMe, R3=OH) → **4-Vinylguaiacol** (R1=R4=R5=H, R2=OMe, R3=OH)

***p*-Coumaric acid** (R1=R2=R4=R5=H, R3=OH) → **4-Vinylphenol** (R1=R2=R4=R5=H, R3=OH)

Caffeic acid (R1=R4=R5=H, R2=R3=OH) → **4-Vinylcatechol** (R1=R4=R5=H, R2=R3=OH)

Supplementary scheme 1 Reaction scheme for CgPAD with different substrates.
