# Supplementary material for: An endogenous factor enhances ferulic acid decarboxylation catalyzed by phenolic acid decarboxylase from Candida guilliermondii
Source: AMB Express. 2012 Jan 4;2:4. doi: 10.1186/2191-0855-2-4 (PMC3402150; doi:10.1186/2191-0855-2-4)
Supplement: Additional file 2 — SDS-polyacrylamide gel electrophoresis of purified enzymes. Supplementary figure 1. [file 2191-0855-2-4-S2.PDF]

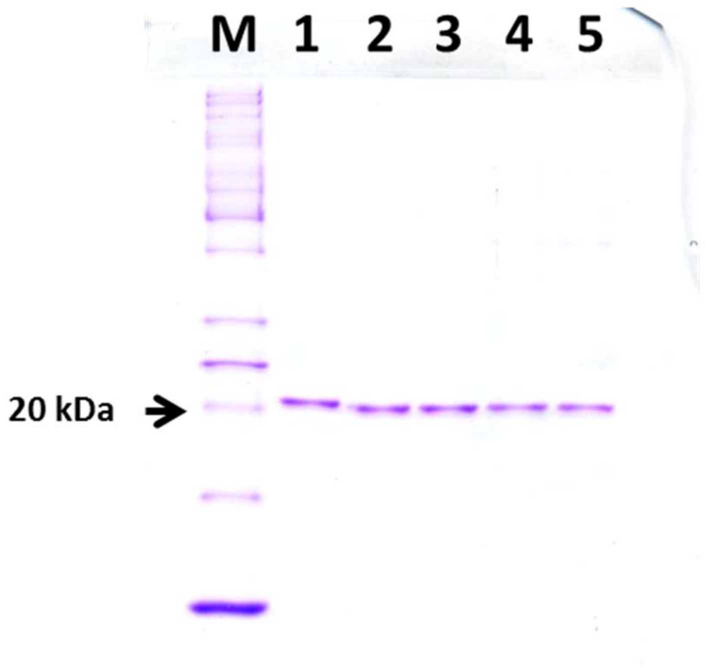

Supplementary figure 1 SDS-polyacrylamide gel electrophoresis of purified enzymes. The electrophoresis was done using a 15% (w/v) acrylamide gel for determination of the molecular masses, using a PageRuler Unstained Protein Ladder kit (Thermo Fisher Scientific, Rockville, MD) as standard markers. Proteins in the gel were stained with Coomassie Brilliant Blue R250. M, Molecular size markers; 1, native enzyme; 2, wild-type enzyme; 3, mutant with M57L; 4, mutant with M57T; 5, mutant with M57A.
