# Supplementary material for: An endogenous factor enhances ferulic acid decarboxylation catalyzed by phenolic acid decarboxylase from Candida guilliermondii
Source: AMB Express. 2012 Jan 4;2:4. doi: 10.1186/2191-0855-2-4 (PMC3402150; doi:10.1186/2191-0855-2-4)
Supplement: Additional file 3 — Primers for CgPAD cDNA cloning. Supplementary table 1. [file 2191-0855-2-4-S3.PDF]

Supplementary table 1 Primers for CgPAD cDNA cloning..

Primer	Sequence (5′ → 3′)
P1*	CARTAYACNTAYGAYAAYGG
P2*	TRTCNCCRTGNGCYTG
P3*	YTCNGGRTGYTCCCARTG
P4	ATATCCAAAATCAACGACACAACGG
P5	CAGTTTCTTCTAACCAATTGACTTGCC
P6	GGCAAGTCAATTGGTTAGAAGAAACTG
P7	CCGTTGTGTCGTTGATTTTGGATAT
P8	ATGTCCTACCAACCACTTATTGG
P9	GAACCTCATTCGCTTCAATTC

* Degenerate primers designed from internal amino acid sequences of native CgPAD.
