# Supplementary material for: An endogenous factor enhances ferulic acid decarboxylation catalyzed by phenolic acid decarboxylase from Candida guilliermondii
Source: AMB Express. 2012 Jan 4;2:4. doi: 10.1186/2191-0855-2-4 (PMC3402150; doi:10.1186/2191-0855-2-4)
Supplement: Additional file 4 — A. Structure-based amino acid sequence alignment of CgPAD with LpPAD of known structure (PDB code 2GC9). B. A model structure of CgPAD incorporating possible catalytic residues Glu82 and Arg60 residues and subsite residues Tyr30 and Tyr32, along with Met57 and Cys66. Supplementary figure 2. [file 2191-0855-2-4-S4.PDF]

A

```
                             α1       β1          β2         β3              β4     β5       β6
LpPAD ------------MTKTFKTLDDFLGTHFIYTYDNGWEYEWYAKNDHTVDYRIHGGMVAGRWVTDQKADIVMLTEGIYKIS    68
CgPAD MSYQPLIGVDAAQVPQEEFDQELKNKHFQYTYDNGWKYEFHVPNDKRIVYSIHGGPMAGRHN-FQTCYYQRVRKNLWQVN    79

              β7         β8       α2                α2                β9
LpPAD WTEPTGTDVALDFMPNEKKLHGTIFFPKWVEEHPEITVTYQNEHIDLMEQSREKYATYPKLVVPEFANITYXGDAGQNNE   148
CgPAD WLEETGTVVSLILDIENKRITTFMAFSQGHWEHPEQAHGDKREDLERWRELSRIGIATNRYLITEQASIDEIFEGRGDLP   159

                 α3
LpPAD DVISEAPYKEMPNDIRNGKYFDQNYHRLNK                                                   178
CgPAD DISLDLPTL---------------------                                                   168
```

B

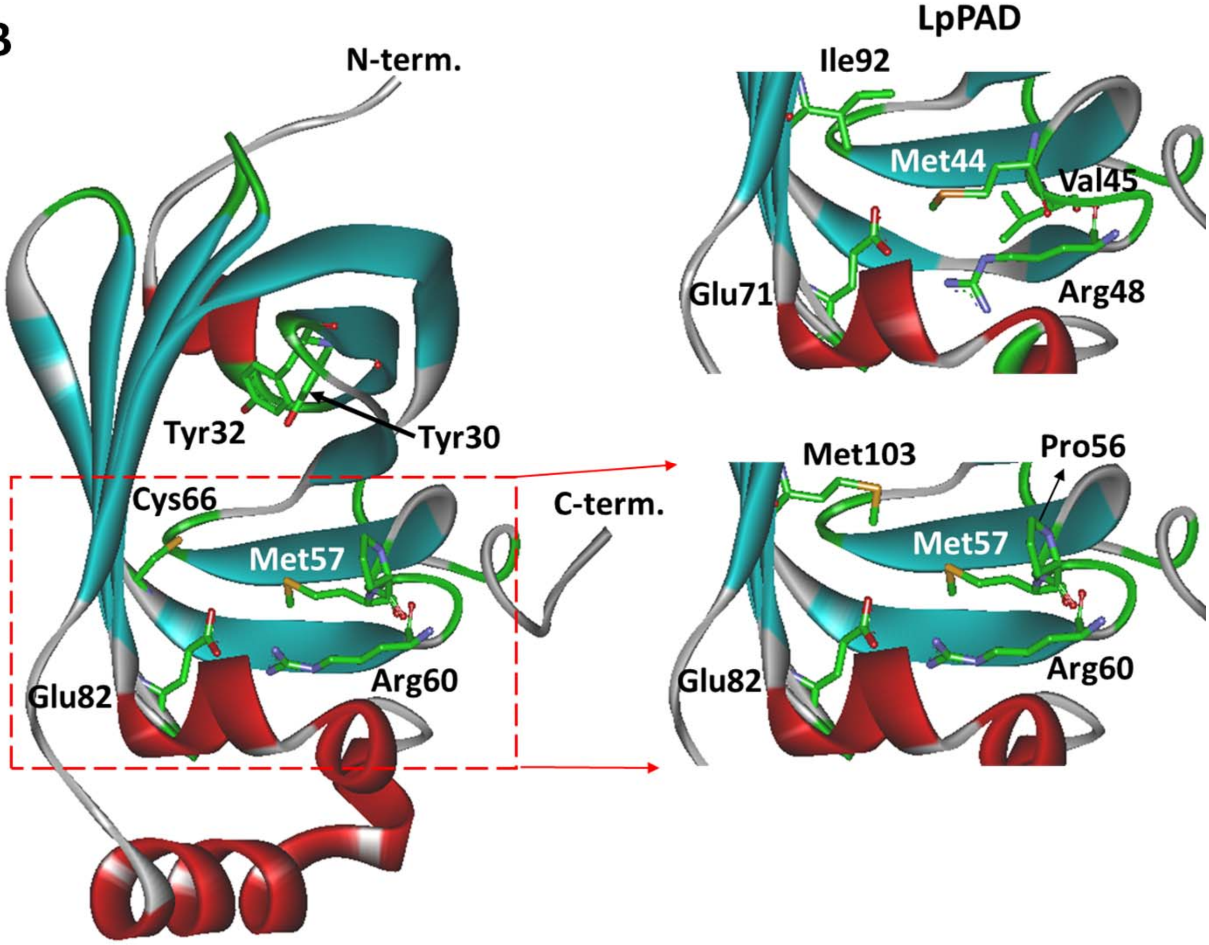

Supplementary figure 2 **A**. Structure-based amino acid sequence alignment of CgPAD with LpPAD of known structure (PDB code 2GC9). The secondary structure prediction of the CgPAD sequence was done by the method of Kabsch and Sander (1983). The helices of LpPAD and CgPAD are shown by lines and β-strands by dotted lines. The methionine residues possibly close to active sites are boxed. **B**. A model structure of CgPAD incorporating possible catalytic residues Glu82 and Arg60 residues and subsite residues Tyr30 and Tyr32, along with Met57 and Cys66. It was constructed with the LpPAD structure as a template. The right plates indicate part of the active-site pocket around Met57 (Val45), Met103 (Ile92), and Pro56 (Met44) in the vicinity of possible catalytic residues Glu82 (Glu71) and Arg60 (Arg48) (amino acids in parentheses correspond to the residues in LpPAD).
