# Supplementary material for: An endogenous factor enhances ferulic acid decarboxylation catalyzed by phenolic acid decarboxylase from Candida guilliermondii
Source: AMB Express. 2012 Jan 4;2:4. doi: 10.1186/2191-0855-2-4 (PMC3402150; doi:10.1186/2191-0855-2-4)
Supplement: Additional file 5 — A. A CPK model of CgPAD. B. Coordination of the catalytic residues in the surrounding Met57 (upper) and Met103 (lower) in the model CgPAD structure. Supplementary figure 3. [file 2191-0855-2-4-S5.PDF]

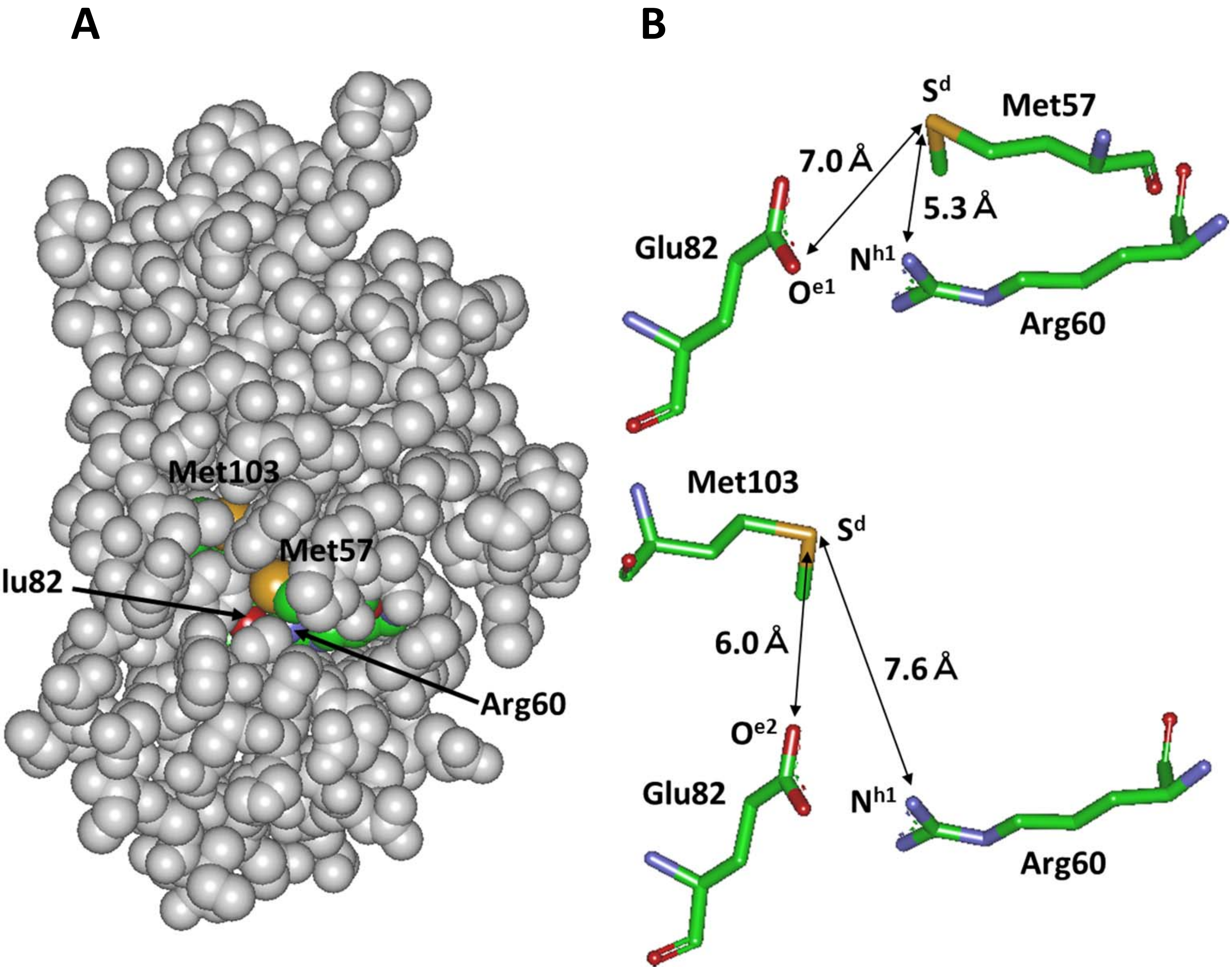

Supplementary figure 3 **A**. A CPK model of CgPAD. The possible catalytic residues Arg60 and Glu82, along with Met57 and Met103, are shown. They are indicated by arrows. Cys66 is located at the other side of the model structure shown. **B**. Coordination of the catalytic residues in the surrounding Met57 (upper) and Met103 (lower) in the model CgPAD structure. The figures are stick models showing the distance from the respective catalytic residues to the S^d atom of either Met57 (upper) or Met103 (lower).
